# Study on clinical characteristics and related factors of schizophrenic patients with intestinal obstruction

**DOI:** 10.1186/s12876-021-02091-y

**Published:** 2022-01-06

**Authors:** Mingchao Li, Ping Guo, Jihua Zeng, Chi Li, Qiuming Ji, Yunqing Zhao, Haiying Chen, Ying Wang, Yunjiao Hu, Lianzhong Liu

**Affiliations:** 1 Department of Psychiatry, Affiliated Wuhan Mental Health Center, Tong Ji Medical College of Huazhong University Science and Technology, Wuhan, 430022 China; 2 Department of Psychiatry, Wuhan Wudong Hospital, Wuhan, 430084 China; 3 Department of Medical, Department of Science, Wuhan Wudong Hospital, No. 46 of Wudong Street, Qingshan District, Wuhan, 430084 China

**Keywords:** Schizophrenia, Intestinal obstruction, Logistic multiple regression analysis, Risk factors, Retrospective study

## Abstract

**Background:**

There are still few studies on the clinical characteristics and related risk factors of schizophrenia patients with intestinal obstruction. Our aim is to explore the clinical characteristics and related risk factors of schizophrenia patients with intestinal obstruction.

**Methods:**

This study focused on schizophrenia patients with intestinal obstruction who were hospitalized in the psychiatric department of a hospital in Wuhan from January 2007 to December 2020 as the main research object. We intend to retrospectively analyze the clinical characteristics and related risk factors of schizophrenia patients with intestinal obstruction.

**Results:**

In the 1937 persons with schizophrenia included in this study, 97 patients were complicated with intestinal obstruction, and the incidence was 5.01%.The results of the study showed that patients with age ≥ 60 years old, visiting time ≥ 24 h, hospital stay ≥ 90 days, history of abdominal surgery, course of disease ≥ 5 years, male, and patients with cardiovascular and cerebrovascular diseases are prone to intestinal obstruction; Logistic multiple regression analysis showed that the related risk factors of schizophrenia patients with intestinal obstruction mainly included the patient's age, visiting time, length of hospital stay, history of abdominal surgery, course of disease and gender.

**Conclusion:**

The older the age, the longer the hospital stay, the longer the course of the disease, the history of previous surgery, and the male schizophrenia who do not see a doctor within 24 h of the onset, the risk of intestinal obstruction is higher, and it is easy to be misdiagnosed and even life-threatening.

## Background

Intestinal obstruction is one of the common acute abdominal diseases in surgery. The main clinical manifestations are abdominal pain, abdominal distension, vomiting and anal exhaust stop [1–3]. Radiographic X-ray plain film of the abdomen shows a liquid–gas plane, and part of the abdominal cavity is gas or dilated. In recent years, researches of domestic and foreign scholars have shown that intestinal obstruction has gradually become a common disease and frequently-occurring disease in patients with mental disorders, especially those with long-term hospitalizations [4–6].

In 2010, Mei Weihong reported the clinical characteristics and measures to improve the curative effect of 6 patients with schizophrenia complicated with acute intestinal obstruction from 2005 to 2009 [7]. Liu Hong published the nursing methods of persons with schizophrenia with intestinal obstruction in 2015 [8]. However, there are still few studies on the clinical characteristics and related risk factors of schizophrenia patients with intestinal obstruction. Therefore, in this study, the persons with schizophrenia with intestinal obstruction in our hospital from January 2017 to January 2020 were selected as the main research subjects, and the clinical characteristics and related risk factors of persons with schizophrenia with intestinal obstruction were retrospectively analyzed, so as to provide more theoretical basis for the prevention and treatment of intestinal obstruction in persons with schizophrenia.

## Methods

### Research subjects

This study is a retrospective study. The main research subjects were 1937 inpatients diagnosed as schizophrenia with intestinal obstruction by the psychiatric department of a hospital in Wuhan from January 2007 to December 2020. The diagnostic criteria for schizophrenia refer to the International Classification of Diseases diagnostic criteria for schizophrenia. The intestinal obstruction met the International Classification of Diseases diagnostic criteria for intestinal obstruction and were confirmed by plain abdominal radiographs.

This study complies with the "Declaration of Helsinki of the World Medical Association" and approved by the ethics committee of Wuhan Wudong Hospital. Written informed consent has been obtained from the guardians of all participants.

### Inclusion and exclusion criteria

Inclusion criteria: (1) Patients with a clear diagnosis of schizophrenia; (2) Age ≥ 18 years; (3) Hospital stay ≥ 60 days; (4) Course of disease ≥ 30 days. Exclusion criteria: (1) patients with other mental illnesses; (2) patients with communication disorders; (3) patients with incomplete case data.

### Main observation indicators

The main observation indicators of this study include the patient's gender, age, visiting time, hospital stay, history of abdominal surgery, and course of disease.

### Statistical analysis

In this study, SPSS 20.0 statistical software is used for data processing, and measurement data are expressed as mean ± standard deviation (x ± s). Counting data is expressed in percentage (%). Logistic regression analysis is used for analysis of related factors, and chi-square test is used for counting data. P < 0.05 indicates that the difference is statistically significant.

## Results

### General information

Among the 1937 patients with schizophrenia included in this study, there were 956 males and 981 females, aged 18–82 years, with an average age of 70.8 ± 6.7 years, and a total of 97 patients had intestinal obstruction.

### Incidence of intestinal obstruction in hospitalized patients with schizophrenia

The results of this study showed that the incidence of intestinal obstruction in the 1937 patients with schizophrenia was 5.01%; Among them, 34 (32.99%) patients with intestinal obstruction were treated non-surgically, and 65 patients (67.01%) with intestinal obstruction received surgical treatment; Eighteen patients with intestinal obstruction were misdiagnosed (18.56%), and 6 patients with intestinal obstruction (6.19%) died.

### Clinical characteristics of acute intestinal obstruction in hospitalized patients with schizophrenia

In this study, among the 1937 hospitalized patients with schizophrenia, patients aged ≥ 60 years, visiting time ≥ 24 h, hospitalization time ≥ 90 days, history of abdominal surgery, course of disease ≥ 5 years, male, and patients with cardiovascular and cerebrovascular diseases are prone to intestines obstruction, see Table 1 for details.

**Table 1:** Comparison of clinical characteristics of acute intestinal obstruction in hospitalized patients with schizophrenia

Related factors	With intestinal obstruction (*n* = 97)		Incidence rate (%)
Age			
< 60 years old	44	1125	2.27^*^
≥ 60 years old	53	715	2.74
Gender			
Male	69	887	3.56^*^
Female	28	953	1.45
Hospital stay			
< 90 days	28	894	1.45^*^
≥ 90 days	69	946	3.56
Course of disease			
< 5 years	35	963	1.81^*^
≥ 5 years	62	877	3.20
Visiting time			
< 24 h	23	1043	3.82^*^
≥ 24 h	74	797	1.19
History of abdominal surgery			
Yes	43	554	2.22^*^
No	54	1286	2.79
History of physical disease			
Yes	50	1032	2.58
No	47	808	2.43

*Compared with the group of without intestinal obstruction, *P* < 0.05

### Logistic multiple regression analysis of intestinal obstruction in hospitalized patients with schizophrenia

Taking the occurrence of intestinal obstruction as the dependent variable and the related factors as the independent variables, after logistic multiple regression analysis, factors such as age, main complaint time, length of hospital stay, history of abdominal surgery, course of disease, gender and other factors are significantly related (*P* < 0.05, see details Table 2.

**Table 2:** Multivariate logistic regression model parameters of intestinal obstruction in hospitalized persons with schizophrenia

Factor	Regression coefficient	Standard error of regression coefficient	Standard regression coefficient	Regression coefficient statistics	P value
Age	1.413	0.316	2.651	24.600	0.000
Clinical time	0.725	0.146	2.457	17.373	0.000
Hospitalization days	1.282	0.423	1.366	10.100	0.001
Abdominal surgery history	1.446	0.379	1.164	8.881	0.003
Course of the disease	0.695	0.216	2.358	7.739	0.005
Gender	2.137	0.843	1.947	4.786	0.029

## Discussion

This study indicates that the incidence of intestinal obstruction in hospitalized schizophrenia patients is 5.01%. Univariate analysis and logistic multiple regression analysis show that the incidence of intestinal obstruction in hospitalized schizophrenia patients is related to the patient’s age, visiting time, length of hospital stay, abdominal surgery history, course of disease, gender, etc. The possible mechanisms are: (1) Lowered sensitivity to pain in patients with schizophrenia [9]. With the increase of age, the body has poor constitution, slow reaction, decreased immunity, decreased conduction function of nervous system, and slow response to pain and stress, especially for the elderly mental patients over 70 years old [10]; (2) Due to the strong sedative effect of anti-schizophrenia drugs, the perception of abdominal pain and distention is slow, and the symptoms and signs at the onset of the disease cannot be reported in time [11–13]; (3) Inpatients with schizophrenia are mostly under closed management. Reduced activity leads to slower gastrointestinal peristalsis. The longer the hospital stay, the greater the chance of intestinal obstruction; (4) Intestinal adhesions are the most common complication after abdominal surgery and the most important risk factor for intestinal obstruction [14, 15], therefore, schizophrenia patients with a history of abdominal surgery are more likely to develop intestinal obstruction; (5) One of the most important risk factors for intestinal obstruction in schizophrenia patients is medication [16]. The anticholinergic effect of drugs for the treatment of schizophrenia can inhibit the contraction of gastrointestinal smooth muscle and reduce gastrointestinal secretion and peristalsis. Therefore, the longer the course of the disease, the longer the medication time, and the greater the dosage, the more frequent the patient’s intestinal obstruction. (6) Male hormones can weaken gastrointestinal smooth muscle contraction and slow gastrointestinal peristalsis by affecting autonomic nerve function, causing male patients with schizophrenia to be more prone to intestinal obstruction than women [17, 18].

In short, the above risk factors are intertwined, causing the patient's intestinal wall to be relaxed and tension-free, the contents of the intestinal cavity cannot move downward, the exhaust and defecation are blocked, the intestinal pressure is increased, resulting in paralytic intestinal obstruction, which makes the patients' feces dry and further aggravate the formation of fecal stones, leading to mechanical intestinal obstruction [19–21]; if it is not detected in time and treated in time during the rounds, it will inevitably be accompanied by various complications and even endanger the life of the patient. Among the 97 cases of intestinal obstruction in this study, 6 patients died, and the mortality rate was 6.19%; 18 patients were misdiagnosed as other diseases, and the misdiagnosis rate was 18.56%.

Schizophrenia is a chronic disease with high disability rate. The patients' knowledge, emotion and intention are not consistent, and their thinking and behavior are abnormal. Long term use of antipsychotics makes the patients slow to respond and will not actively and correctly reflect and describe their physical discomfort. Their poor compliance with clinical physical examination and treatment has brought great difficulties to the diagnosis and treatment of intestinal obstruction in schizophrenia. In order to detect and treat schizophrenia with intestinal obstruction early, it should be strengthened from the following aspects: (1) Carefully observe and ask the patient's diet and stool conditions, and promptly treat the patient with constipation could prevent ileus [22]; (2) Actively carry out comprehensive work and entertainment treatment, behavior correction treatment and improve the cognitive function of patients, if there is discomfort in patients, take the initiative to tell the doctor about the condition; (3) Keep on doing appropriate exercises such as gymnastics every day, and do gardening therapy for rehabilitation training [23] to alleviate the adverse effects of antipsychotic drugs and sedation, despite some difficulties; (4) For patients with a history of abdominal surgery, physical examination should be carried out every day, especially abdominal visual and tactile percussion [24]; (5) For patients with schizophrenia who are hospitalized for a long time and have a long course of disease, timely adjust the dose of antipsychotic drugs and maintain a lower dose of maintenance therapy drugs [25]. Dose reduction of antipsychotics may alleviate persistent constipation [26]; (6) For male patients, strengthen the control of active substances such as tobacco and alcohol, and regularly test various biochemical indicators [27].

This research still has the following shortcomings. First of all, this study is a retrospective study, not a randomized controlled experiment, so there is still a certain risk of bias. Secondly, this study is a single-center clinical study, and subsequent multi-center clinical studies are still needed for further discussion. Finally, the sample size included in this study is relatively small, and it is still necessary to increase the sample size for further research.

## Conclusion

The older the age, the longer the hospital stay, the longer the course of the disease, the history of previous surgery, and the male schizophrenia who did not seek medical attention within 24 h of the onset, the risk of intestinal obstruction is higher, and it is easy to be misdiagnosed and even life-threatening.

## Data Availability

The datasets used and/or analysed during the current study are available from the corresponding author on reasonable request.
